# NUTRITION MANAGEMENT OF INFLAMMATORY BOWEL DISEASES: A CONSENSUS OF THE BRAZILIAN ORGANIZATION FOR CROHN’S AND COLITIS (GEDIIB)

**DOI:** 10.1590/S0004-2803.24612026-042

**Published:** 2026-08-10

**Authors:** Daniéla Oliveira MAGRO, Carina ROSSONI, Raquel Rocha, Ligia Yukie SASSAKI, Marcello IMBRIZI, Isadora Sayuri Macedo TUMA, Carina Andriatta BLUME, Cristina Henschel MATOS, Ryan Nunes Yoshio YOSHIHARA, Maria Izabel Lamounier de VASCONCELOS, Patricia Hanako Ribeiro SATO, Maria Paula Carlin CAMBI, Claiza Barretta LA BELLA, Matheus Freitas Cardoso de AZEVEDO, Julio Maria Fonseca CHEBLI, Cristiane Kibune NAGASAKO, Júlio Pinheiro BAIMA, Claudio Saddy Rodrigues COY, Paulo Gustavo KOTZE, Cristina FLORES, Rogério SAAD-HOSSNE, Eduardo Garcia VILELA

**Affiliations:** 1 Universidade Estadual de Campinas (Unicamp), Campinas, SP, Brasil.; 2 Universidade de Lisboa, Faculdade de Medicina, Instituto de Saúde Ambiental, Lisboa, Portugal.; 3 Universidade Federal da Bahia, Escola de Nutrição, Salvador, BA, Brasil.; 4 Universidade Estadual Paulista, Faculdade de Medicina de Botucatu, Botucatu, SP, Brasil.; 5 Pontifícia Universidade Católica do Paraná, Curitiba, PR, Brasil; 6 DIIMUNO - Centro de Tratamento de Doenças Inflamatórias Intestinais e Imunomediadas, Porto Alegre, RS, Brasil.; 7 Clínica privada, Palhoça, SC, Brasil; 8 Associação Brasileira de Colite e Doença de Crohn, São Paulo, SP, Brasil.; 9 Universidade Federal de Santa Catarina, Hospital Universitário, Santa Catarina, SC, Brasil.; 10 Universidade do Vale do Itajaí, Itajaí, SC, Brasil.; 11 Universidade de São Paulo, Hospital das Clínicas, São Paulo, SP, Brasil.; 12 Universidade Federal de Juiz de Fora, Juiz de Fora, MG, Brasil.; 13 Universidade Federal de Minas Gerais, Hospital das Clínicas, Ebserh, Instituto Alfa de Gastroenterologia, Belo Horizonte, MG, Brasil.

**Keywords:** Inflammatory bowel disease, adults, nutrition management, dietary therapy, Doenças inflamatórias intestinais, adultos, manejo nutricional, terapia nutricional

## Abstract

**Background:**

Inflammatory bowel diseases (IBD), specifically Crohn’s disease and ulcerative colitis, are multifactorial conditions. Genetic and behavioral factors are associated with disease development and influence its progression. It is known that diet plays a role in this pathogenic process.

**Objective:**

to position nutritional and dietary factors in the management of IBD.

**Methods:**

This consensus was developed by nutritionists, gastroenterologists, and colorectal surgeons, members of GEDIIB (Brazilian Organization for Crohn’s disease and colitis). A systematic review of the most recent evidence was conducted to support the recommendations/statements. All recommendations and statements were endorsed by stakeholders and IBD experts in a modified Delphi panel, achieving a consensus rate of at least 80%.

**Results and conclusion:**

the recommendations were directed according to disease activity and severity. Nutritional screening and diagnosis, nutritional therapy in the active and remission phases of the disease, dietary management and exclusion diets, and supplementation were addressed. The consensus is targeted at nutritionists, general practitioners, gastroenterologists, and surgeons interested in managing adults with IBD. It supports decision-making by health insurance companies, regulatory agencies, and health institutional managers.

## HIGHLIGHTS

• First consensus on nutritional management in IBD of the GEDIIB.

• Nutritional management is part of IBD treatment in the induction and maintenance of remission.

• Dietary patterns across different Crohn´s disease phenotypes during active disease and clinical remission.

• Dietary patterns across different ulcerative colitis phenotypes during active disease and clinical remission.

## RESUMO

**Contexto:**

As doenças inflamatórias intestinais (DII), em especial a doença de Crohn e a colite ulcerativa, são condições multifatoriais. Fatores genéticos e comportamentais estão associados ao desenvolvimento da doença e influenciam sua progressão. Sabe-se que a dieta desempenha papel fundamental nesse processo patogênico.

**Objetivo:**

Posicionar os fatores nutricionais e dietéticos no manejo da DII.

**Métodos:**

Este consenso foi desenvolvido por nutricionistas, gastroenterologistas e cirurgiões colorretais, membros do GEDIIB (Organização Brasileira de Doença de Crohn e Colite). Foi realizada uma revisão sistemática das evidências mais recentes para fundamentar as recomendações/declarações. Todas as recomendações e declarações incluídas foram endossadas em um painel Delphi modificado por partes interessadas e especialistas em DII, com taxa de consenso de pelo menos 80%.

**Resultados e conclusão:**

As recomendações foram direcionadas à atividade e à gravidade da doença. Foram abordados a triagem e o diagnóstico nutricional, a terapia nutricional nas fases ativa e de remissão da doença, o manejo dietético e as dietas de exclusão, além da suplementação. O consenso é voltado a nutricionistas, clínicos gerais, gastroenterologistas e cirurgiões interessados no manejo de adultos com DII. Ele serve como suporte à tomada de decisão por operadoras de planos de saúde, agências reguladoras e gestores de instituições de saúde.

## INTRODUCTION

The relevance of nutritional management in inflammatory bowel diseases (IBD) has been well documented, supported by robust studies on the prevention, induction, and maintenance of remission. Given the rising prevalence of IBD in Brazil^1^, specialized training for nutritionists and healthcare professionals involved in patient care has become imperative to ensure that clinical practice is grounded in evidence-based practice. In view of this, the Brazilian Organization for Crohn´s and Colitis (GEDIIB) organized the first consensus on the nutrition management of IBD.

This multidisciplinary consensus aimed to engage multiple stakeholders, including policymakers, government agencies, private health insurance providers, and diverse physicians and nutritionists. Among these, the document is also directed to those who do not routinely manage IBD, for whom it may have a greater practical impact and support clinical decision-making.

## METHODS

This consensus provides relevant guidance for decision-making in the nutritional management of IBD. It synthesizes recommendations based on scientific evidence and the most up-to-date knowledge. Expert consensus, especially in the healthcare field, can synthesize current evidence to inform clinical practice, management, research, and health policy, while maintaining diversity and independence of opinion, decentralization, and the specialization of knowledge.

The consensus was aimed at nutritionists and health professionals interested in the treatment and nutritional management of adults with IBD. A panel of 22 experts comprised 11 nutritionists, 8 gastroenterologists, and 3 surgeons specializing in IBD, all members of GEDIIB.

 The methodological basis of the GEDIIB-guided Nutrition in IBD was a formal systematic review that addressed 10 clinical questions and was stratified into 24 items of interest. Each question, its eligibility criteria, search strategies used, and the summary of included studies are specified in SUPPLEMENTARY MATERIAL. These systematic searches, conducted by a professional librarian (completed June 2025), used Medline, Embase, ClinicalTrials.gov, Google Scholar, and a manual search of references. The primary outcomes extracted were clinical rather than intermediate and varied according to the clinical question. Whenever feasible, outcomes were expressed as the absolute risk of events in each comparator group, the risk difference, 95% confidence intervals (95%CI), and the number needed to treat (NNT), or the number needed to harm (NNH). Risk of bias was assessed using the RoB 2 tool for randomized trials and the ROBINS-I tool for observational studies. The certainty of the evidence was evaluated using the GRADE framework. According to GRADE, the quality of evidence is classified as **High quality**: further research is very unlikely to change our confidence in the estimate of effect; **Moderate quality**: further research is likely to have an important impact on our confidence in the estimate of effect and may change the estimate; **Low quality**: further research is very likely to have an important impact on our confidence in the estimate of effect and is likely to change the estimate; **Very low quality**: any estimate of effect is very uncertain^2^. When common outcomes were identified across selected studies, data were synthesized via meta-analysis using fixed or random-effects models, depending on the heterogeneity calculated by the I^2^ statistic. Finally, a summary of findings was provided to support the clinical recommendations addressing each initial research question. All the steps were monitored, evaluated, and validated by a GEDIIB panel of specialists.

All statements underwent three rounds of voting and panel members’ feedback, followed by nine rounds of revisions. The second round of voting was extended to all members, with an opportunity for feedback, followed by a single revision round. Panel members met online in February 2026 for the final discussion and consensus voting. Consensus was defined as >80% agreement, and only statements that met this threshold were included.

## SUPLEMMENTARY MATERIAL

**QUESTION 1.1**

**Diet has an important role in the pathogenesis of inflammatory bowel disease (IBD), both ulcerative colitis (UC) and Crohn’s disease (CD). Epidemiological studies indicate that adopting a Western diet (low in fruits and vegetables, rich in fats, ω-6 fatty acids, red meat, and processed foods) contributes to the rising incidence of IBD in developing countries. Was a higher intake of Ultra-processed food associated with higher IBD?**

**Eligibility criteria - Question 1.1**

P: Adults.

I: Western diet or ultra-processed food.

O: Incidence of IBD (UC and CD).

Study design: cohort.

Consulted period: no restriction.

Language: Portuguese, Spanish, English. 

**Search strategy - Question 1.1**

#1 (Inflammatory Bowel Diseases OR Inflammatory Bowel Disease OR Colitis, Ulcerative OR Ulcerative Colitis OR Crohn Disease OR Crohn’s Enteritis OR Crohn’s Disease OR Crohn’s Disease)

#2 (Diet, Western OR Western Diets OR Western Dietary OR Occidental Diet OR Occidental Diets OR Western Diet OR Meat-Sweet Diet OR Meat-Sweet Diets)

#3 (Food, Processed OR Processed Food OR Processed Foods)

#4 (Red Meat OR Red Meat OR Beef OR Lamb Meat OR Lamb Meats OR Pork Meats OR Pig Meat OR Pig Meats OR Pork OR Bacon OR Cured Ham) 

#5 (Diet, High-Fat OR High-Fat Diet OR High-Fat Diets OR High Fat Diet OR High Fat Diets)

#6 = #1 AND (#2 OR (#3 OR #4 OR #5)) = FINAL

**Results - Question 1.1**

A total of 1.028 articles were retrieved from Medline: 976, Embase: 51, Scholar: 1, and 8 publications were selected to support this assessment. 

**QUESTION 1.2**

**Diet has an important role in the pathogenesis of inflammatory bowel disease (IBD), both ulcerative colitis (UC) and Crohn’s disease (CD). Do food additives have a role in the development of IBD?**

**Eligibility criteria - Question 1.2**

P: Adults.

I: Food additives (salt, sweet, maltodextrins, emulsifiers, coating agents, stabilizers, carrageenan).

O: Incidence of IBD (UC and CD).

Study design: cohort, case-control, cross-sectional, or Clinical Trial 

Consulted period: no restriction. Language: Portuguese, Spanish, English. 

**Search strategy - Question 1c**

#1 (Inflammatory Bowel Diseases OR Inflammatory Bowel Disease OR Colitis, Ulcerative OR Ulcerative Colitis OR Crohn Disease OR Crohn’s Enteritis OR Crohn’s Disease OR Crohn’s Disease)

# 2 (Food Additive OR Food Additives OR Flavoring Agent OR Flavor Additives OR Flavor Additive OR Flavor Enhancers OR Flavor Enhancer OR Flavoring Agents OR Sweetening Agents OR Sweetening Agent OR Sweeteners OR Sweetener OR Sugar Substitutes OR Sugar Substitute OR Dietary Sucrose OR Emulsifying Agents OR Emulsifying Agent OR Emulsifiers OR Dextrins OR Maltose OR Carrageenan OR Carrageenin OR Salt OR maltodextrins OR coating agents OR stabilizers)

Final = #1 AND #2

**Results - Question 1.2**

A total of 3.327 articles were retrieved from Medline: 3.268, Embase: 40, Scholar: 19, and 5 publications were selected to support this assessment.

**QUESTION 2.1**

**Diet has an important role in the pathogenesis of inflammatory bowel disease (IBD), both ulcerative colitis (UC) and Crohn’s disease (CD). Does vitamin D play a protective role in the natural history of IBD?**

**Eligibility criteria - Question 2.1**

P: Adults.

I: Vitamin D.

O: Protective role in the natural history of IBD (UC and CD).

Study design: cohort, case-control, cross-sectional, or clinical trials. 

Consulted period: no restriction. Language: Portuguese, Spanish, English. 

**Search strategy - Question 1d**

#1 (Inflammatory Bowel Diseases OR Inflammatory Bowel Disease OR Colitis, Ulcerative OR Ulcerative Colitis OR Crohn Disease OR Crohn’s Enteritis OR Crohn’s Disease OR Crohn’s Disease)

#2 (Vitamin D OR Cholecalciferol OR Hydroxycholecalciferols OR Ergocalciferols OR (25 Hydroxyvitamin D2) OR Dihydrotachysterol OR Calcifediol OR Dihydroxycholecalciferols OR (24,25-Dihydroxyvitamin D3) OR Calcitriol)

Final = #1 AND #2

**Results - Question 2.1**

A total of 1,272 articles were retrieved from Medline (1,230), Embase (37), and Scholar (5), of which 1 was selected to support this assessment.

**QUESTION 2.2**

**Diet has an important role in the pathogenesis of inflammatory bowel disease (IBD), both ulcerative colitis (UC) and Crohn’s disease (CD). May breastfeeding have a protective role in the development of IBD?**

**Eligibility criteria - Question 2.2**

P: Adults.

I: Breastfeeding.

O: Protective role in the development of IBD (UC and CD).

Study design: cohort, case-control, cross-sectional, or systematic review.

Consulted period: no restriction.

Language: Portuguese, Spanish, English. 

**Search strategy - Question 2.2**

#1 (Inflammatory Bowel Diseases OR Inflammatory Bowel Disease OR Colitis, Ulcerative OR Ulcerative Colitis OR Crohn Disease OR Crohn’s Enteritis OR Crohn’s Disease OR Crohn Disease)

#2 (Breastfed OR Breastfeeding OR Breast Fed OR Breast Feeding)

FINAL: 1 AND #2

**Results - Question 2.2**

A total of 452 articles were retrieved (Medline: 403, Embase: 117, Scholar: 32), and 4 publications were selected to support this assessment.

**QUESTION 2.3**

**Diet has an important role in the pathogenesis of inflammatory bowel disease (IBD), both ulcerative colitis (UC) and Crohn’s disease (CD). May the Mediterranean diet have a protective role in the development of IBD?**

**Eligibility criteria - Question 2.3**

P: Adults.

I: Mediterranean diet.

O: Protective role in the development of IBD (UC and CD).

Study design: cohort, case-control, cross-sectional, or clinical trial 

Consulted period: no restriction.

Language: Portuguese, Spanish, English. 

**Search strategy - Question 1f**

#1 (Inflammatory Bowel Diseases OR Inflammatory Bowel Disease OR Colitis, Ulcerative OR Ulcerative Colitis OR Crohn Disease OR Crohn’s Enteritis OR Crohn’s Disease OR Crohn’s Disease)

#2 (Diet, Mediterranean OR Mediterranean Diet OR Mediterranean Diets)

FINAL: 1 AND #2

**Results - Question 2.3**

A total of 126 articles were retrieved (Medline: 112, Embase: 6, Scholar: 8), and 2 publications were selected to support this assessment.

**QUESTION 3.1**

**Patients with IBD are at higher risk of malnutrition; hence, all patients with IBD should be screened for malnutrition at presentation. Does the prevalence of malnutrition in patients with IBD depend upon disease subtype, severity, extent, and duration?**

**Eligibility criteria - Question 3.1**

P: Adults with IBD

I: disease subtype, severity, extent, and duration.

O: Risk of malnutrition.

Study design: observational studies.

Consulted period: no restriction.

Language: Portuguese, Spanish, English. 

**Search strategy - Question 3.1**

(Inflammatory Bowel Diseases OR Inflammatory Bowel Disease OR Colitis, Ulcerative OR Ulcerative Colitis OR Crohn’s Disease OR Crohn’s Enteritis OR Crohn’s Disease OR Crohn’s Disease) AND (Malnutrition OR “Nutritional Deficiency” OR “Nutritional Deficiencies” OR Undernutrition OR Malnourishment OR Malnourishments OR Protein Deficiency OR “Nutrition Disorder” OR “Nutrition Disorders”)

**Results - Question 3.1**

A total of 4.094 articles were retrieved (Medline: 3.917, Embase: 98, Scholar: 79, and 12 publications were selected to support this assessment.

**QUESTION 3.2**

**Patients with IBD are at higher risk of malnutrition; hence, all patients with IBD should be screened for malnutrition at presentation. Is body mass index alone sufficient for nutritional assessment of a patient with IBD?**

**Eligibility criteria - Question 3.2**

P: IBD

I: Body mass Index (alone)

C: Other measurements 

O: Nutritional assessment or Malnutrition diagnosis

Study design: observational studies.

Consulted period: no restriction.

Language: Portuguese, Spanish, English.

**Search strategy - Question 3.2**

(Inflammatory Bowel Diseases OR Inflammatory Bowel Disease OR Colitis, Ulcerative OR Ulcerative Colitis OR Crohn’s Disease OR Crohn’s Enteritis OR Crohn’s Disease OR Crohn’s Disease) AND (Body Mass Index OR BMI)

**Results - Question 3.2**

A total of 2.185 articles were retrieved (Medline: 1.866, Embase: 262, Scholar: 57), and 4 publications were selected to support this assessment.

**QUESTION 4.1**

**Patients with IBD are at higher risk of malnutrition; hence, all patients with IBD should be screened for malnutrition at presentation. Plasma proteins, such as albumin, should not be used as markers of nutrition in active disease?**

**Eligibility criteria - Question 4.1**

P: IBD

I: Plasma proteins (such as albumin)

C: Other measurements 

O: Nutritional assessment or Malnutrition diagnosis

Study design: observational studies.

Consulted period: no restriction.

Language: Portuguese, Spanish, English. 

**Search strategy - Question 4.1**

(Inflammatory Bowel Diseases OR Inflammatory Bowel Disease OR Colitis, Ulcerative OR Ulcerative Colitis OR Crohn’s Disease OR Crohn’s Enteritis OR Crohn’s Disease OR Crohn’s Disease) AND (Protein OR Proteins OR Lipoproteins OR Glycoproteins OR Globulins OR Blood Proteins OR Albumin OR Albumins) AND (Nutritional OR Nutrition)

**Results - Question 4.1**

A total of 6.183 articles were retrieved (Medline: 4.287, Embase: 1.305, Scholar: 591), and after an initial selection of 42 studies, 3 pieces of evidence were selected to support this assessment.

**QUESTIONS 4.2**

Should patients with inflammatory bowel disease (IBD) receive adequate calories, proteins, and fats in their diet? Is the calorie and protein requirement of a patient with IBD in remission like that of a healthy individual? However, protein requirements increase in patients with active diseases. Is an oral diet with high protein (to 1.2-1.5 g/Kg/d in adults) recommended for active inflammation in IBD?

**Eligibility criteria - Questions 4.2**

P: Adults with IBD

I: Protein intake

O: Clinical remission; Clinical improvement; Disease status

Study design: Randomized Clinical Trials, cohort, cross-sectional, case-control

Consulted period: No restriction.

Language: No restriction 

**Search strategy - Questions 4.2**

(Inflammatory Bowel Diseases OR Inflammatory Bowel Disease OR Colitis, Ulcerative OR Ulcerative Colitis OR Crohn’s Disease OR Crohn’s Enteritis OR Crohn’s Disease OR Crohn’s Disease) AND (Proteins OR Protein) AND (Diet OR Diets OR Intake OR Ingestion)

**Results - Questions 4.2**

The search carried out until August 2024 retrieved, after duplicates removal, 3567 articles (Medline: 2528; Embase: 714; CENTRAL: 307; Lilacs: 18). In agreement with the eligibility criteria, one study was included to support this assessment.

**QUESTION 5.1**

Is any dietary item established to cause relapses of disease activity in a patient in remission?

**Eligibility criteria - Question 5.1**

P: Inflammatory Bowel Disease in remission

I: Dietary 

O: Relapse, Gastrointestinal Symptoms

Study design: RCT, Cohort, Cross-sectional, Case-Control

Consulted period: No restriction.

Language: No restriction

**Search strategy - Question 5.1**

This question will be answered based on search strategies already used for other questions.

**Results - Question 5.1**

The search carried out retrieved, after duplicates removal, 3011 articles (Medline: 1407; Embase: 1404; CENTRAL: 174; Lilacs: 26). In agreement with the eligibility criteria, 5 were included to support this assessment. A second search carried out retrieved, after duplicates removal, 273 articles (Medline: 123; Embase: 146; CENTRAL: 66; Lilacs: 8). In agreement with the eligibility criteria, 6 were included to support this assessment. Two RCT’s compared patients in a low-FODMAP diet and a normal diet. Four cohorts compared patients before and after a low-FODMAP diet.

**QUESTION 5.2**

Should milk not be routinely restricted to all patients with IBD lactose intolerance?

**Eligibility criteria - Question 5.2**

P: Inflammatory Bowel Disease in remission

I: Milk restrictions

O: Relapse, Gastrointestinal Symptoms

Study design: RCT, Cohort, Case-Control

Consulted period: No restriction.

Language: No restriction

**Search strategy - Question 5.2**

(Inflammatory Bowel Diseases OR Inflammatory Bowel Disease OR Colitis, Ulcerative OR Ulcerative Colitis OR Crohn’s Disease OR Crohn’s Enteritis OR Crohn’s Disease) AND (Dairy OR Milk OR Lactose)

**Results - Question 5.2**

The search carried out retrieved, after duplicates removal, 3011 articles (Medline: 1407; Embase: 1404; CENTRAL: 174; Lilacs: 26). In agreement with the eligibility criteria, 3 were included to support this assessment.

**QUESTION 5.3**

A low FODMAP diet may help in alleviating irritable bowel syndrome (IBS)-like symptoms associated with IBD?

**Eligibility criteria - Question 5.3**

P: Adults with irritable bowel syndrome (IBS)-like symptoms associated with IBD.

I: low-FODMAP diet

O: Symptoms relief

Study design: Randomized Clinical Trials, Cohort

Consulted period: No restriction.

Language: No restriction

**Search strategy - Question 5.3**

(Inflammatory Bowel Diseases OR Inflammatory Bowel Disease OR Colitis, Ulcerative OR Ulcerative Colitis OR Crohn’s Disease OR Crohn’s Enteritis OR Crohn’s Disease) AND (FODMAP).

**Results - Question 5.3**

The search carried out until September 2024 retrieved, after duplicates removal, 273 articles (Medline: 123; Embase: 146; CENTRAL: 66; Lilacs: 8). In agreement with the eligibility criteria, 2 were included to support this assessment. 

**QUESTION 6.1**

Should supplementation with n-3 fatty acids, antioxidants, and curcuma be advised to support maintenance of remission in patients with IBD?

**Eligibility criteria - Question 6.1**

P: IBD in remission

I: Glutamine, n-3 fatty acids, antioxidants, and curcuma

O: Disease activity

Study design: RCT, cohort, case-control

Consulted period: No restriction.

Language: No restriction

**Search strategy - Question 6.1**

(Inflammatory Bowel Diseases OR Inflammatory Bowel Disease OR Colitis, Ulcerative OR Ulcerative Colitis OR Crohn Disease OR Crohn’s Enteritis OR Crohn’s Disease OR Crohn’s Disease) AND (Glutamine OR N 3 Fatty Acid OR N 3 Fatty Acids OR Antioxidants OR Antioxidant OR Anti-Oxidant OR Anti-Oxidants OR Curcuma OR Turmeric OR Curcumin OR Curcuminoids) AND (Diet OR Diets OR Supplements OR Supplement OR Supplementation OR Supplementations).

**Results - Question 6.1**

The search carried out until October 2024 retrieved, after duplicates removal, 1477 articles (Medline: 1098; Embase: 214; CENTRAL: 161; LILACS: 4). In agreement with the eligibility criteria, 3 RCTs were included to support this assessment.

**QUESTION 6.2**

Should curcumin be used as an adjunct therapy in the treatment of patients with active ulcerative colitis to induce remission?

**Eligibility criteria - Question 6.2**

P: Adults with Ulcerative Colitis

I: Curcumin

O: Clinical remission; Clinical improvement

Study design: Randomized Clinical Trials

Consulted period: No restriction.

Language: No restriction

**Search strategy - Question 3c**

(Inflammatory Bowel Diseases OR Inflammatory Bowel Disease OR Colitis, Ulcerative OR Ulcerative Colitis OR Crohn Disease OR Crohn’s Enteritis OR Crohn’s Disease OR Crohn’s Disease) AND (Curcuma OR Turmeric OR Curcumin OR Curcuminoids)

**Results - Question 6.2**

The search carried out until August 2024 retrieved, after duplicates removal, 633 articles (Medline: 401; Embase: 175; CENTRAL: 57; Lilacs: 0). In agreement with the eligibility criteria, 6 were included to support this assessment

**QUESTION 7.1**

**More evidence is required before elimination diets, such as the Crohn’s disease exclusion diet (CDED), for IBD patients.**

**Eligibility criteria - Question 7.1**

P: Inflammatory Bowel Disease in remission

I: Elimination diets (SCD, CDED, semi-vegetarian, anti-IBD diet)

O: Relapse, Gastrointestinal Symptoms

Study design: RCT, Cohort, Case-Control

Consulted period: No restriction.

Language: No restriction

**Search strategy - Question 7.1**

(Inflammatory Bowel Diseases OR Inflammatory Bowel Disease OR Colitis, Ulcerative OR Ulcerative Colitis OR Crohn Disease OR Crohn’s Enteritis OR Crohn’s Disease) AND (Eliminations Diet OR Elimination Diets OR Exclusion Diet OR Exclusion Diets OR Specific carbohydrate diet OR Crohn’s disease exclusion diet OR CDED OR semi-vegetarian diet OR Flexitarian OR IBD Anti-Inflammatory Diet)

**Results - Question 7.1**

The search carried out until October 2024 retrieved, after removing duplicates, 1,354 articles (Medline: 939; Embase: 138; CENTRAL: 277). In agreement with the eligibility criteria, 2 were included to support this assessment.

**QUESTION 7.2**

**EEN is effective in adult CD, but is it inferior to corticosteroids inducing remission?**

**Eligibility criteria - Question 7.2**

P: Crohn’s disease 

I: Exclusive enteral nutrition 

C: Corticosteroids 

O: Induction of remission 

Patients included were adults with Crohn’s Disease. There was no restriction on study design, period, or language.

**Search strategy - Question 7.2**

(Crohn Disease OR Crohn’s Enteritis OR Crohn’s Disease OR Crohn’s Disease) AND (Enteral OR EEN OR Polymeric OR Elemental OR Semi-elemental OR Formula) AND (Corticosteroids OR Glucocorticoids OR Prednisolone OR Prednisone).

**Results - Question 7.2**

A total of 906 articles were retrieved (Medline: 426, Embase: 278, CENTRAL: 200, LILACS: 1). 02 articles were assessed to support this assessment.

**QUESTION 7.3**

Is there no difference between elemental and polymeric formulae regarding efficacy? Should a standard formula be used (without glutamine, n-3 fatty acids, antioxidants, or immunonutrients)?

**Eligibility criteria - Question 7.3**

P: Crohn’s disease and UC (adult)

I: Elemental diet 

C: Polymeric formulae

O: Efficacy OR harm OR induction of remission 

Study design: No restriction. 

Consulted period: No restriction.

Language: English, Spanish, Portuguese, Italian

**Search strategy - Question 7.3**

(Inflammatory Bowel Diseases OR Inflammatory Bowel Disease OR Colitis, Ulcerative OR Ulcerative Colitis OR Crohn’s Disease OR Crohn’s Enteritis OR Crohn’s Disease OR Crohn’s Disease) AND (Enteral OR EEN OR Polymeric OR Elemental OR Formula) AND (Nutrition OR Nutritional OR Feed OR Feeding OR Tube OR Diet)

**Results - Question 7.3**

A total of 3782 articles were retrieved (Medline: 2413, Embase: 738, CENTRAL: 523, LILACS: 108). Three were used to support this assessment.

**QUESTION 7.4**

Has partial enteral nutrition (PEN) been documented to be useful for maintenance of remission in luminal CD along with pharmacotherapy?

**Eligibility criteria - Question 7.4**

P: Crohn’s disease and UC (adult)

I: Partial Enteral Nutrition 

C: Enteral Nutrition OR other nutrition 

O: Efficacy OR maintenance of remission 

Study design: No restriction. 

Consulted period: No restriction. 

Language: No restriction. 

**Search strategy - Question 7.4**

(Inflammatory Bowel Diseases OR Inflammatory Bowel Disease OR Colitis, Ulcerative OR Ulcerative Colitis OR Crohn Disease OR Crohn’s Enteritis OR Crohn’s Disease OR Crohn’s Disease) AND (Enteral OR Tube)

**Results - Question 7.4**

A total of 5059 articles were retrieved (Medline: 2341, Embase: 1055, CENTRAL: 1555, LILACS: 108). Four were used to support this assessment.

**QUESTION 8.1**

**Intravenous iron should be considered as first-line treatment in patients with clinically active IBD, those with previous intolerance to oral iron, those with hemoglobin below 10 d/dL, and in patients who need erythropoiesis-stimulating agents. Is the goal of iron supplementation to normalize the hemoglobin levels and iron stores?**

**Eligibility criteria - Question 8.1**

P: IBD + anemia

I: Intravenous iron

C: Oral iron

O: Hb increase, safety

Study design: randomized clinical trials

Consulted period: No restriction.

Language: No restriction 

**Search strategy - Question 8.1**

(Inflammatory Bowel Diseases OR Inflammatory Bowel Disease OR Ulcerative Colitis OR Crohn Disease OR Crohn’s Enteritis OR Crohn’s Disease OR Crohn’s Disease) AND (iron OR ferric OR ferrous)

**Results - Question 8.1**

The search carried out until January 2025 retrieved, after duplicates removal, 3425 articles (Medline: 1588; Embase: 1568; CENTRAL: 245; Lilacs: 24). In agreement with the eligibility criteria, 4 were included to support this assessment.

**QUESTION 8.2**

Proactive screening for osteopenia and osteoporosis, and its treatment, should be done according to current osteoporosis guidelines. In IBD patients (adults) with active disease and those who are steroid-treated, serum calcium and 25(OH) vitamin D should be monitored and supplemented if required to help prevent low bone mineral density?

**Eligibility criteria - Questions 8.2**

P: Inflammatory Bowel Disease

I: (Screening and treatment for osteopenia and osteoporosis) OR Screening and supplementations of serum calcium and 25(OH) vitamin D

O: Fractures

Study design: Randomized controlled trials, cohorts

Consulted period: No restriction.

Language: No restriction 

**Search strategy - Questions 8.2**

(Inflammatory Bowel Diseases OR Inflammatory Bowel Disease OR Ulcerative Colitis OR Crohn Disease OR Crohn’s Enteritis OR Crohn’s Disease) AND (Metabolic Bone Disease OR Osteopenia OR Low Bone Density OR Osteoporosis OR Calcium OR Vitamin D)

**Results - Question 8.2**

The search carried out until January 2025 retrieved, after duplicates removal, 9549 articles. In agreement with the eligibility criteria, 1 cohort was included to support this assessment.

**QUESTION 9.1**

**If the nutritional goals cannot be met with an oral diet alone, oral nutritional supplements (ONS) or enteral nutrition (EN) should be initiated prior to surgery/perioperative phase?**

**Eligibility criteria - Question 9.1**

P: Inflammatory Bowel Disease

I: ONS / EN

C: No ONS / EN

O: Postoperative outcomes and complications

There were no restrictions for study design, period, and language.

**Search strategy - Question 9.1**

(Inflammatory Bowel Diseases OR Inflammatory Bowel Disease OR Ulcerative Colitis OR Crohn’s Disease OR Crohn’s Enteritis OR Crohn’s Disease) AND (Enteral Nutrition OR Oral Nutritional Supplement OR Oral Nutritional Intervention OR Oral Supplement OR Oral Nutritional Supplements OR Oral Nutritional Interventions OR Oral Nutritional Supplementation OR ONS)

**Results - Question 9.1**

A total of 9819 articles were retrieved after duplicates removal (Medline: 2602, Embase: 6934, CENTRAL: 267, LILACS: 16). Six articles were assessed to support this assessment.

**QUESTION 9.2**

Is the use of PN in the perioperative period reserved for patients who are unable to tolerate EN?

**Eligibility criteria - Question 9.2**

P: Inflammatory Bowel Disease

I: Preoperative Parenteral Nutrition

C: No PN

O: Postoperative outcomes and complications

There was no restriction on study design, period, or language. 

**Search strategy - Question 9.2**

(Inflammatory Bowel Diseases OR Inflammatory Bowel Disease OR Ulcerative Colitis OR Crohn’s Disease OR Crohn’s Enteritis OR Crohn’s Disease) AND (Parenteral Nutrition OR Parenteral Feeding OR Intravenous Feeding)

**Results - Question 9.2**

A total of 3462 articles were retrieved (Medline: 1591, Embase: 1809, CENTRAL: 44, LILACS: 18). Two articles were assessed to support this assessment.

**QUESTION 9.3**

Should the ERAS (early/enhanced recovery after surgery) protocol be followed in the perioperative period?

**Eligibility criteria - Question 9.3**

P: Inflammatory Bowel Disease

I: ERAS 

C: No ERAS

O: Postoperative outcomes and complications

Only studies that applied the ERAS protocol were included; other enhanced recovery protocols were not included. There was no restriction on study design, period, or language. 

**Search strategy - Question 9.3**

(Inflammatory Bowel Diseases OR Inflammatory Bowel Disease OR Ulcerative Colitis OR Crohn Disease OR Crohn’s Enteritis OR Crohn’s Disease) AND (ERAS OR Enhanced Recovery After Surgery OR Enhanced Postsurgical Recovery OR Enhanced Postsurgical Recoveries)

**Results - Question 9.3**

A total of 239 articles were retrieved (Medline: 116, Embase: 73, CENTRAL: 50, LILACS: 0). Two articles were assessed to support this assessment, one RCT and one retrospective cohort.

**QUESTION 10.1**

**Undernutrition, overnutrition, and altered body composition are predictors of poor postoperative outcomes in surgical IBD patients.**

**Eligibility criteria - Question 10.1**

P: Inflammatory Bowel Disease

I: Undernutrition, overnutrition, and altered body composition. 

O: Postoperative outcomes and complications

There was no restriction on study design, period, or language. 

**Search strategy - Question 10.1**

(Inflammatory Bowel Diseases OR Inflammatory Bowel Disease OR Ulcerative Colitis OR Crohn’s Disease OR Crohn’s Enteritis OR Crohn’s Disease) AND (Undernutrition OR Overnutrition OR Body Composition)

**Results - Question 10.1**

A total of 4192 articles were retrieved after duplicate removal (Medline: 3520, Embase: 436, CENTRAL: 72, LILACS: 164). Two articles were assessed to support this assessment.

**QUESTION 10.2**

**Is Sarcopenia common among CD patients awaiting surgery, and is it a risk factor for adverse postoperative outcomes and complications?**

**Eligibility criteria - Question 10.2**

P: Crohn’s disease 

I: Sarcopenia 

C: No sarcopenia 

O: Adverse postoperative outcomes and complications

Patients included were adults with Crohn’s Disease. 

There was no restriction on study design, period, and language. 

**Search strategy - Question 10.2**

(Inflammatory Bowel Diseases OR Inflammatory Bowel Disease OR Crohn’s Disease OR Crohn’s Disease) AND (Sarcopenia)

**Results - Question 10.2**

A total of 395 articles were retrieved (Medline: 208, Embase: 271, CENTRAL: 14, LILACS: 6). Three articles were assessed to support this assessment.

**QUESTION 10.3**

Can teduglutide and intestinal transplantation be considered in individual CD patients with Short Syndrome Bowel (SBS) or Intestinal Failure (IF) when Parenteral Nutrition therapy fails?

**Eligibility criteria - Question 10.3**

P: CD patients with SBS or IF

I: Teduglutide and/or intestinal transplantation

O: Improvement of nutritional status

There was no restriction on study design, period, or language. 

**Search strategy - Question 10.3**

(Inflammatory Bowel Diseases OR Inflammatory Bowel Disease OR Crohn Disease OR Crohn’s Enteritis OR Crohn’s Disease) AND (Intestine Transplantation OR Intestinal Transplantation OR Small Bowel Transplantation OR Teduglutide)

**Results - Question 10.3**

A total of 2819 articles were retrieved after duplicates removal (Medline: 2479, Embase: 79, CENTRAL: 135, LILACS: 126). One article was assessed to support this assessment.

### Dietary patterns and IBD pathogenesis

### Western diet (low in fruits and vegetables, high in fats, ω-6 fatty acids, red and processed meats, and ultra-processed foods)

**Recommendations**

A Western-type dietary pattern is associated with an increased risk of developing Crohn’s disease (CD) and, to a lesser extent, ulcerative colitis (UC)^3^ ^-^ ^7^.

High intake of red and processed meat is associated with an increased risk of ulcerative colitis^3^ ^,^ ^6^.

Current evidence is insufficient and inconsistent to support or refute an association between high fiber intake or low meat consumption and the risk of late-onset IBD^5^.

Higher consumption of ultra-processed foods (UPF) is associated with increased CD incidence, with conflicting evidence for UC^6^ ^,^ ^8^.

**Consensus: 85.71%**

**Low quality of evidence**

Diet is recognized as a potential environmental factor in the pathogenesis of IBD, including both CD and UC. A dietary pattern characterized by high intake of snacks, prepared meals, non-alcoholic beverages, and sauces, along with minimally processed foods, has been associated with IBD risk^3^ ^-^ ^5^. However, an extensive study did not support a significant association between high fiber intake and/or low meat intake and the lower risk of late-onset IBD^5^. In the context of dietary patterns, UK Biobank data, which collected genetic and clinical information from half a million participants, show that total Fatty Acids (FAs), including saturated FAs, polyunsaturated FAs, monounsaturated FAs, and omega-6 FAs, were not associated with IBD risk. On the other hand, Omega-3 FAs (docosahexaenoic acid) can protect against UC, as was observed with unprocessed/minimally processed foods, which were associated with a lower risk of CD^6^. While one study found a higher intake of UPF was associated with an increased risk of incident CD but not UC^8^, another investigation found a positive association with both conditions^9^; however, a third study failed to report such an association^10^. The most striking finding was an increased relative risk of both CD and UC associated with fast-food consumption^7^. Although the data are conflicting, the recommendation to avoid a Western-type diet is warranted.

### Consumption of food additives (such as emulsifiers, sugar-sweetened beverages, artificial sweeteners, and salt)

**Recommendation**

Certain food additives, including emulsifiers, sugar-sweetened, artificial sweeteners, and salt, may contribute to the pathogenesis of IBD^11^ ^-^ ^15^. Evidence remains inconsistent, and causality cannot be confirmed.

**Consensus: 95.24%**

**Low quality of evidence**

Dietary patterns before disease onset are similar between patients with CD and UC, showing increased consumption of sweetened beverages, processed and fatty meats, fried foods, salt, industrial desserts (e.g., ice cream), mayonnaise, and reduced intake of seeds, nuts, and yogurt^11^. Consumption of artificial sweeteners aligns with healthy preferences among IBD patients^12^. Patients with CD were more frequently exposed to processed foods and food additives than healthy controls, suggesting a potential role in both disease onset and perpetuation of inflammation^13^. Specifically, higher intakes of aspartame, sucralose, and the emulsifier polysorbate-80 have been reported among CD patients compared with controls^14^. Findings regarding beverages indicate an association between sugar-sweetened beverages and IBD risk, whereas artificially sweetened beverages and natural fruit juices have not shown a consistent association^14^ ^,^ ^15^. Nevertheless, whether high sugar and salt intake is a causal factor or a secondary phenomenon related to disease onset remains uncertain. Although the data appear preliminary, the recommendation to avoid certain food additives is justified. Excessive UPF intake is associated with reduced populations of beneficial bacteria, decreased butyrate production (essential for maintaining intestinal barrier integrity), and possibly an increased risk of CD^16^.

**Prevention of IBD onset**

### Vitamin D

**Recommendation**

Current evidence does not support a protective or causal role of vitamin D levels in the development of IBD^17^.

**Consensus: 85.71%**

**Low quality of evidence**

The protective effect of vitamin D in the natural history or development of IBD is controversial. A Mendelian randomization study was conducted to examine 120,013 individuals from the Copenhagen City Heart Study, the Copenhagen General Population Study, and a Copenhagen-based cohort of patients with IBD, incorporating genetic data from 408,455 individuals in the UK Biobank, including 1,707 CD cases and 3,147 UC cases. The multivariable-adjusted hazard ratios per 10 nmol/L increase for 25-hydroxyvitamin D level were 1.04 (95%CI: 0.93 to 1.16) for CD and 1.13 (95%CI: 1.06 to 1.21) for UC. Genetically, plasma 25-hydroxyvitamin D level was also not associated with the risk of CD or UC^17^. These findings suggest no causal or protective relationship between vitamin D levels and the risk of IBD onset.

### Breastfeeding

**Recommendations**

Evidence is insufficient to establish breastfeeding as a protective factor against IBD. Observational data suggest a possible reduction in the risk of CD and UC with longer breastfeeding duration (≥6-12 months)^18^ ^-^ ^21^.

Given the heterogeneity of study designs and inconsistent findings, no causal association between breastfeeding duration and IBD development can be confirmed^18^ ^-^ ^20^.

**Consensus: 80.95%**

**Very low quality of evidence**

Breastfeeding has been associated with protection from IBD, but some controversy remains in the scientific literature. A population-based case-control study in Israel (n=2,789; birth years 1964-1976) found no difference in IBD risk between breastfed and non-breastfed individuals^22^. Conversely, other case-control studies associated the absence or shorter duration of breastfeeding with an increased risk of IBD: Lack of breastfeeding: UC OR 1.5 (95%CI 1.1-2.1); CD OR 1.9 (95%CI 1.1-3.3)^18^. Breastfeeding <6 months**:** CD OR 2.7 (95%CI 1.7-4.4); UC OR 1.7 (95%CI 1.02-2.8)^19^. In the Swiss IBD cohort (1,111 IBD patients and 352 controls), breastfeeding duration did not differ between CD and UC, but controls reported shorter breastfeeding (<6 months) than IBD patients (*P*=0.014). Breastfeeding <6 months was associated with a decreased risk of UC and indeterminate colitis (OR 0.47; 95%CI 0.28-0.81; *P*=0.006)^23^. A retrospective comparative study in Belgium and Romania found breastfeeding to be a protective factor for Belgian IBD patients (OR 0.31; 95%CI 0.10-0.90)^20^. A systematic review and meta-analysis including 14 studies demonstrated a protective association between breastfeeding and the development of both CD and UC in pediatric and adult-onset disease. A dose-dependent effect was observed, with the most significant reduction in risk when breastfeeding continued ≥12 months: CD**:** OR 0.20 (95%CI 0.08-0.50), UC**:** OR 0.21 (95%CI 0.10-0.43)^21^. Overall, although some heterogeneity exists across studies, the preponderance of evidence suggests a potential protective effect of breastfeeding against the development of IBD, particularly for longer durations (>6-12 months).

### Mediterranean diet

**Recommendation**

Adherence to a Mediterranean diet (MD) has been associated with a lower risk of IBD, particularly CD^24^ ^,^ ^25^. However, evidence regarding UC is limited and insufficient to confirm a causal protective effect^25^.

**Consensus: 85.71%**

**Low quality of evidence**

The MD is scientifically recognized for its metabolic benefits and improvement of the gut microbiota^24^ ^,^ ^25^. However, limited evidence exists regarding IBD prevention, and more studies are needed on maintaining IBD remission. The data from the Nurses’ Health Study (1986-2014), Nurses’ Health Study II (1991-2015), and Health Professionals follow-up study (1986-2014), which assess lifestyles with serial questionnaires observed individual associations between 5 healthy lifestyle factors after IBD diagnosis (never smoking, body mass index 18.5-24.9 kg/m^2^, vigorous physical activity, alternate Mediterranean diet score ≥4, and light drinking [0.1-5.0 g/d])^24^. Among individual associations, the MD (HR, 0.69; 95%CI, 0.49-0.98) was protective against IBD^24^. In another prospective cohort of Swedish men and women (n=83,147; age range: 45-79 years), a validated food frequency questionnaire was used to calculate an adherence score to a modified MD^25^. A higher MD score was associated with a lower risk of CD (*P*=0.03) but not with UC (*P*=0.61). The rate of poor adherence to an MD (score=0-2) was 27% in these cohorts, corresponding to a population-attributable risk of 12% for later-onset CD^25^. A typical MD is characterized by a predominance of plant foods (fruits, vegetables, cereals with as little processing as possible, pulses, nuts, and seeds), with moderate amounts of dairy products, mostly fermented (cheese and yogurt); low to moderate amounts of fish (especially oily, omega-3 rich types) and poultry; and low amounts of red meat^26^. Consequently, it may be recommended to reduce the risk of IBD as a therapeutic nutritional tool.

### Nutrition assessment in IBD

### Malnutrition in IBD

**Recommendations**

The prevalence of malnutrition increases with disease activity and severity in both CD and UC^27^ ^,^ ^28^.

Malnutrition is more frequent in patients with low body mass index (BMI)^29^, prior intestinal surgery^30^, active inflammation, and complicated disease phenotypes, including strictures, fistulas, and ileostomy^31^ ^,^ ^32^.

In UC, malnutrition is more commonly associated with extensive and active disease^33^ ^,^ ^34^, whereas in CD, it is associated with ileocolonic involvement and severe disease^33^ ^,^ ^35^.

Undernutrition is associated with worse clinical outcomes, including increased rates of hospitalization, surgery, and venous thromboembolism events^30^ ^,^ ^36^.

**Consensus: 100.00%**

**Low quality of evidence**

Malnutrition (especially undernutrition) is highly prevalent among patients with IBD and increases with disease activity, hospitalization, and complications^27^. Patients with a BMI < 25 kg/m^2^ had a higher disease activity (*P*<0.05) and less appetite (*P*=0.03)^29^. More severe disease was associated with a higher likelihood of undernutrition at diagnosis in both CD and UC^28^. Another study found that malnutrition was associated with underweight, active disease, and increased hospital admissions^30^, and disease activity and undernutrition were associated with poorer quality of life (QOL)^37^. In CD, ileocolonic location, moderate-to-severe activity, strictures or fistulas, current steroid use, and shorter disease duration were independent risk factors for malnutrition^33^. In UC, malnutrition at diagnosis was significantly more frequent among patients with more extensive disease and active flare, and the risk of malnutrition decreased with age^33^ ^,^ ^34^. Conversely, UC patients in clinical or histological remission had body composition similar to that of controls without IBD^38^. Reduced subcutaneous adiposity index (SAI), visceral adiposity index (VAI), and skeletal muscle mass index (SMI) were also associated with greater disease activity and more complex behavior^35^. Among hospitalized UC patients, the prevalence of malnutrition was lower in those with histological remission (grades 0-1) than in those with histological activity (grades 2-4) (*P*=0.001). Based on the Controlling Nutritional Status (CONUT) score, 70.3% of patients were malnourished, predominantly mild (59.9%), with 9.3% moderate and 1.1% severe malnutrition^34^. The manifestations of malnutrition in patients with IBD were diverse and related to the severity of the disease. A retrospective cohort study of 73 adult patients with IBD (48 CD and 25 UC) found that 65.75% of patients had nutritional risk according to the Nutritional Risk Screening (NRS-2002), and 58.90% were identified as malnourished according to the Global Leadership Initiative on Malnutrition (GLIM) criteria. The GLIM includes reduced muscle mass as one of the phenotypic criteria for diagnosing malnutrition^30^. The most strongly associated factors with malnutrition were disease recurrence, prior IBD surgery, diarrhea, and ileostomy^31^ ^,^ ^32^. The proportion of patients treated with surgery was significantly higher in the malnourished group compared to the non-malnourished group. In a large community-based cohort, malnutrition was linked to higher risks of hospitalization, surgery, and venous thromboembolism event (VTE). Compared with patients at low-risk according to Malnutrition Universal Screening Tool (MUST), those at medium or high malnutrition risk had increased hazard ratios for: Hospitalization: aHR 1.80 (95%CI 1.34-2.42) and 1.90 (95%CI 1.30-2.78); Surgery: aHR 2.28 (95%CI 1.60-3.26) and 2.38 (95%CI 1.52-3.73), and VTE: aHR 2.79 (95%CI 1.33-5.59) respectively^36^. Malnutrition correlates with disease activity, extent, and complicated behavior, but not with disease duration or location consistently^32^. Nutritional care is clearly important in the treatment of patients with IBD and includes the prevention and management of undernutrition.

### Body mass index (BMI)

**Recommendations**

**BMI is widely used for nutritional screening but lacks sensitivity to detect loss of muscle mass and changes in body composition; therefore, it is insufficient for comprehensive nutritional assessment in IBD patients** ^39^ **.**

**Expert Opinion**

A comprehensive nutritional assessment, including evaluation of body composition (e.g., fat-free mass index [FFMI], skeletal muscle index [SMI], dual-energy X-ray absorptiometry [DEXA]) and functional measures (e.g., handgrip strength), is recommended to accurately identify malnutrition and sarcopenia in IBD patients^40^ ^-^ ^43^.

**Consensus: 85.71%**

**Low quality of evidence**

While BMI is widely used to track nutritional status (undernutrition, eutrophic status, and overweight/obesity) according to World Health Organization (WHO) criteria^44^, it is not an accurate measure of body composition^39^. It lacks sensitivity in predicting the loss of body cell and skeletal muscle mass. The handgrip strength index is an effective and convenient parameter for predicting the functional nutritional status and muscular health of CD patients^41^. Other methods, such as DEXA Absorptiometry (DXA), identify many patients with reduced skeletal mass index (SMI) who are not detected by standard methodologies^42^. The SMI could detect reductions in muscular mass that the body mass index could not measure^43^. The detection rate of malnutrition using FFMI (85.1%) was markedly higher than using BMI. Human body composition analysis can identify patients with reduced muscular mass that are not detected by commonly used nutritional assessment scales/parameters^45^. Patients with CD had more impaired anthropometric and body-composition indicators than those with UC^46^. Therefore, additional assessment of body composition (DEXA, bioelectrical impedance analysis, ultrasound, computed tomography, and magnetic resonance imaging) and muscle function (grip strength) is essential in routine clinical care to diagnose low FFMI and initiate intervention promptly^40^ ^,^ ^41^. Waist circumference is an indicator of visceral adiposity and a nutritional assessment measure to consider^40^.

### Indicators of nutritional status

### Albumin in active IBD

**Recommendations**

Serum albumin and other plasma proteins should not be used as isolated markers of nutritional status in patients with active IBD, as they are influenced by inflammation and disease activity^47^ ^,^ ^48^.

Despite this limitation, low serum albumin has important prognostic relevance. It is associated with nonresponse to therapy, increased need for colectomy, and higher rates of postoperative complications in patients with IBD^49^.

**Expert opinion**

Prealbumin may reflect protein malnutrition and can be considered as part of nutritional assessment, particularly when interpreted in the context of inflammatory activity^47^.

**Consensus: 80.95%**

**Very low-quality evidence**

Disease-related malnutrition (DRM) and micronutrient deficiencies, including low levels of hemoglobin, albumin, and prealbumin, are common in IBD and correlate with disease activity and hospitalization rates^47^. However, serum albumin concentration is influenced by inflammation, hydration status, and hepatic synthesis, making it an unreliable marker of nutritional status, particularly during active disease^47^. Albumin reflects disease activity more than actual nutrient stores and should therefore not be used in isolation to diagnose or monitor malnutrition in IBD. Despite this limitation, low serum albumin holds prognostic value. In a cohort from resource-limited settings, hypoalbuminemia significantly predicted primary nonresponse (PNR) in UC and PNR or secondary loss of response (SLR) in CD to anti-TNF therapy^48^. Furthermore, in the context of acute severe UC, a low albumin (≤2.5 g/dL) combined with elevated band neutrophils (≥13%) demonstrated a 100% positive predictive value for colectomy within 90 days^48^. In surgical patients, preoperative hypoalbuminemia was an independent risk factor for postoperative complications in CD (even among individuals with normal BMI), indicating its utility as a risk stratification biomarker, though not as a nutritional measure^49^. While plasma proteins such as albumin may predict disease severity and outcomes, they are unreliable nutritional markers in active IBD.

### Protein intake during remission and active IBD

**Recommendations**

In patients with IBD in remission, oral protein intake appears to be comparable to that of healthy individuals^50^.

Current evidence is insufficient to demonstrate increased protein requirements in patients with active IBD compared with healthy individuals.

**Expert opinion**

There is no direct, consistent evidence to support a universally increased protein requirement for all patients with active IBD. However, in clinical contexts characterized by active inflammation, hypercatabolism, malnutrition, loss of lean body mass, or corticosteroid use, a higher protein intake may be required, typically 1.2-1.5 g/kg/day^51^.

**Consensus: 90.48%**

**Very low-quality evidence**

In a case-control study comparing 61 IBD patients (31 active; 30 in remission) with 101 healthy controls, healthy individuals had higher total protein intake (99.5 [74.3-134.0] vs 77.5 [70.5-87.4] g/day; *P*=0.001), although the percentage of energy from protein was similar between groups (*P*=0.171)^50^. Within the IBD cohort, patients in remission consumed a higher proportion of dietary protein than those with active disease (19% vs 17.3%; *P*=0.024), while the absolute protein intake (g/kg and total) did not differ significantly (*P*=0.422)^50^. Overall, these findings suggest that protein intake in IBD remission is comparable to that of healthy individuals, and there is no consistent evidence supporting increased protein requirements in active disease.

### Nutritional components and activity in IBD

Daily milk, gluten, and FODMAP (fermentable oligosaccharides, disaccharides, monosaccharides, and polyols)

**Recommendations**

Current evidence does not support an association between milk^52^ ^-^ ^54^ or gluten^55^ consumption and disease relapses in patients with IBD in remission.

Low-FODMAP diets do not appear to influence IBD activity; however, they may be beneficial for the management of functional gastrointestinal symptoms, such as bloating and abdominal pain^56^.

**Consensus: 80.95%**

**Low quality of evidence**

**Expert Opinion**

A low FODMAP diet should not be routinely prescribed for patients with IBD who are malnourished or at nutritional risk, due to its restrictive nature and its potential negative impact on energy and protein intake as well as overall nutritional status^57^.

**Consensus: 95.00%**

Data are heterogeneous on whether dairy consumption aggravates IBD symptoms. Variation in daily milk consumption did not significantly affect IBD symptoms or relapse rates. Most studies show that dairy consumption is not associated with disease activity, relapses, or symptom worsening^52^ ^-^ ^54^. Regarding a gluten-free diet, patients reported that their symptoms improved, likely due to increased fermentation products resulting from dysbiosis. A gluten-free diet did not improve endoscopic or histo-endoscopic scores or fecal calprotectin levels after 8 weeks^55^. Following the same premise, the low FODMAP diet was studied in IBD patients with irritable bowel syndrome (IBS). It was compared to a regular diet, but it remained unclear whether the low FODMAP diet provides significantly greater relief from IBS-like symptoms in IBD patients in remission. In CD, there was no difference in the Harvey-Bradshaw Index score between the low FODMAP diet (3.2±0.4) and the control diet (3.4±0.5; *P*=0.814) at the end of the trial. Similarly, no significant differences in the partial Mayo score in UC were observed between the low FODMAP diet (0.2±0.2) and the control diet (0.2±0.2; *P*=0.951) and in fecal calprotectin levels at the end of the trial between the low FODMAP diet (60.0±9.4 μg/g) and the control diet (59.6±9.8 μg/g, *P*=0.976). Serum CRP concentrations also did not differ significantly. However, the low FODMAP diet is more effective in alleviating non-specific symptoms, such as bloating, than a standard diet^56^.

### FODMAP and IBS-like symptoms

**Recommendations**

A low-FODMAP diet may relieve IBS-like symptoms in patients with IBD in remission or quiescent disease^56^ ^,^ ^58^.

Current evidence does not support the use of a low-FODMAP diet to control IBD activity or improve inflammatory outcomes when compared with a regular diet.

**Consensus: 80.95%**

**Low quality of evidence**

A higher proportion of IBD patients reported adequate relief of gut symptoms following the low FODMAP diet (14/27, 52%) than the control diet (4/25, 16%, *P*=0.007). Patients had a greater reduction in IBS severity scores following the low FODMAP diet (mean decrease of 67±78) than following the control diet (mean reduction of 34±50). However, this difference was not significant (*P*=0.075). Following the low FODMAP diet, patients had higher health-related quality-of-life scores (81.9±1.2) than those on the control diet (78.3±1.2; *P*=0.042). A targeted analysis revealed that, in stool samples collected at the end of the study period, patients on the low FODMAP diet had significantly lower abundances of *Bifidobacterium adolescentis, Bifidobacterium longum*, and *Faecalibacterium prausnitzii*, considered symbionts, than patients on the control diet. However, microbiome diversity and markers of inflammation did not differ significantly between groups. Compared to a regular diet, it remains unclear whether the low-FODMAP diet provides considerably greater relief from IBS-like symptoms in IBD patients in remission^56^. In IBD patients with quiescent disease or those in remission, the low-FODMAP diet shows improvement from baseline in reducing IBS-like symptoms^58^. See also recommendation 5.1.

### Dietary supplementation in IBD

### Antioxidants, omega-3, and curcumin for induction and maintenance of remission in IBD

**Recommendation 1**

Current evidence does not support the efficacy of antioxidant (59) or omega-3 fatty acid supplementation for the induction or maintenance of remission in CD or UC; therefore, their routine use is not recommended^60^ ^,^ ^61^.

**Consensus: 85.71%**

**Low quality of evidence**

**Recommendation 2**

Curcumin in combination with mesalamine may contribute to achieving remission in patients with mild-to-moderate UC; however, current evidence is inconsistent and insufficient for routine use^62^ ^,^ ^67^.

**Consensus: 85.71%**

**Low quality of evidence**

One clinical trial randomized 57 patients with CD to receive vitamin E (800 IU) plus C (1000 mg) (n=28) or placebo (n=29) for 4 weeks. At baseline, the Crohn’s Disease Activity Index (CDAI) between groups was similar (121±18 vs 138±18; *P*>0.05). After 4 weeks, there were no significant changes in CDAI score (137±24 vs 136±20; *P*>0.05). Supplementation with vitamin C and E did not differ from placebo in disease activity^59^. In relation to omega-3 FAs supplementation, a study randomized patients with CD to receive omega-3 FAs (fish oil containing eicosapentaenoic acid and docosahexaenoic acid) (n=70) or placebo (n=65) for 1 year. Patients receiving supplementation experienced 38 relapses, compared to 34 in the placebo group. The difference in relapse-free intervals between the two groups was not statistically significant (*P*=0.38)^60^. Another RCT included 96 UC patients; at baseline, 56 were in relapse, and 40 were in remission. Patients were randomized to either fish oil or olive oil^.^ During the trial, 69 patients achieved remission, 35 with fish and 34 with olive oil. In line with these findings, another study found no difference in relapse rates between groups. The proportion of patients with relapses was 42% in the fish oil group and 43% in the placebo group after 6 months, and 54% vs 63% after 1 year (*P*=0.44)^61^. Omega-3 fatty acid supplementation (fish oil) did not differ from placebo in relapse-free intervals or relapse^59^ ^-^ ^61^.

Curcumin is an Asian root from the ginger family, with anti-inflammatory and antioxidant properties. The curcumin type, associated drugs, doses, and follow-up period varied across studies^62^ ^,^ ^63^. A randomized, double-blind, controlled trial compared the effects of 450 mg of oral curcumin with mesalamine 2.4 g, placebo, and mesalamine 2.4 g in patients with UC of mild to moderate severity for 8 weeks. It concluded that a low dose of curcumin (450mg/day) was ineffective in inducing remission of UC^63^. Another randomized trial using high doses of curcumin (3g/day) associated with 4 g of mesalamine was associated with 53.8% of clinical remission at week 4, compared with none of the patients receiving placebo (OR 42, 95%CI, 2.3-7.60, *P*=0.01), and 38% in the curcumin group achieved endoscopic remission, compared with none of placebo group (OR 20,7, 95%CI, 1.1-3.93, *P*=0.043). Adding curcumin to mesalamine therapy was superior to mesalamine alone, achieving clinical and endoscopic remission in patients with mild-to-moderate active UC^62^. In other clinical outcome studies, the efficacy data were inconsistent, with results ranging from significant differences to no difference relative to placebo. Patients received different formulations, durations, and doses of curcumin as adjunct therapy for UC^64^ ^-^ ^67^.

### Dietary therapy for induction and maintenance of remission in IBD

### Crohn’s Disease Exclusion Diet (CDED)

**Recommendation**

Current evidence for exclusion diets, including the Crohn’s Disease Exclusion Diet (CDED), is limited, and these diets should not be routinely recommended in patients with IBD^11^ ^,^ ^68^.

**Consensus 95.00%**

**Low quality of evidence**

Literature offers limited studies on the application of exclusion diets (CDED) in patients with CD. Some of them address the disease in remission, and the experience of isolated dietary exclusion in patients with active disease, as a clinical trial, has not been reported in the literature. Among these, 24 patients were advised to follow the diet to maintain remission. Within this subgroup, 10 patients (41.4%) used the CDED exclusively, 6 (25%) as an adjunct therapy, 2 (8.3%) as a bridging therapy, and 6 (25%) combined it with steroids or antibiotics. After 12 weeks on the CDED, 20 patients (83.3%) remained in remission. With an average follow-up of 6.8 months (±6.5), 5 patients (20.8%) required additional therapeutic interventions^11^. CDED may improve remission rates across multiple CD presentations and indications^68^. However, the lack of a control group limits the strength of the conclusions. An exclusion diet that reduces disaccharides, saturated fats, emulsifiers, and red or ultra-processed meats has not demonstrated superior outcomes compared with a regular diet, suggesting that its benefits in remission maintenance may be similar to those achieved with standard dietary practices^11^. See also recommendations 1.1 and 1.2.

### Exclusive enteral nutrition (EEN)

**Recommendation**

EEN may be effective for inducing remission in adults with CD. Limited and heterogeneous evidence suggests that its efficacy may be comparable to that of corticosteroids; however, this comparison should be interpreted with caution^69^ ^,^ ^70^.

**Consensus: 80.95%**

**Low quality of evidence**

EEN is a nutritional treatment in which all foods are replaced with an enteral nutritional formula for 6 or 8 weeks. The evidence is more robust for EEN nutrition therapy in inducing remission in children with mild-to-moderate CD. EEN can be considered in pediatric DC patients, in adult patients who can tolerate or make nasogastric tube feeding, or when steroid side effects are not tolerated or better avoided^71^. A clinical trial with 32 patients with active CD was conducted to determine the efficacy and safety of polymeric enteral nutrition compared with steroids in achieving and maintaining clinical remission. The polymeric diet was administered via a nasogastric tube via pump-assisted continuous infusion [2800 ± 120 kcal/day]. The steroid group received 1 mg/kg/day of prednisone. Both treatments effectively induced clinical remission: 12 of the 15 patients assigned to the polymeric diet and 15 of the 17 patients given steroids achieved clinical remission (defined by a Van Hees index <120) within 4 weeks of treatment. It was also observed that point scores decreased in both groups over a four-week treatment period, by 34.8 (4.9%) for steroids and 32.3 (5%) for enteral nutrition (a difference of 2.5%; [95%CI -11.8% to +16.8%]). The mean time to achieve remission was similar in both groups (2.0 (1) vs 2.4 (1.2) weeks)^69^. In another study, 16 patients were randomly assigned to the EEN and corticosteroid groups. While EEN has a more pronounced effect on nutritional indicators, corticosteroids have a greater impact on inflammatory indicators^70^.

### Elemental and polymeric enteral nutrition

**Recommendation 1**

Elemental and polymeric enteral nutrition formulas appear to have similar efficacy in inducing remission in patients with active CD^72^ ^-^ ^74^.

**Consensus: 80.95%**

**Recommendation 2**

Standard polymeric formulas (without added glutamine, omega-3 fatty acids, antioxidants, or immunonutrients) are appropriate for enteral nutrition therapy^72^.

**Consensus: 90.48%**

**Very low quality of evidence**

In a randomized, double-blind trial that involved 21 patients, of whom 11 received polymeric diet (PD) and 10 elemental diets (ED), the reduction in CDAI after treatment with PD (303±27 to 97±11) was similar to that observed with ED (359±67 to 112±19). Similar changes in C-reactive protein (CRP) were also observed (62±20 to 9±6 and 16±5 to 4±1.6, respectively). Enteral nutrition effectively treats active CD, regardless of whether the diet is polymeric or elemental. Both are equally effective^72^. Another prospective randomized clinical trial included 30 patients with active CD unresponsive to steroids and/or malnourished. It sought to determine whether an ED or a defined polymeric formula for 4 to 6 weeks would be more effective. Both groups showed similar improvements in nutritional status, biological inflammation, alpha-1 antitrypsin clearance, and colonoscopy lesions. Relapses occurred within a year after discharge in most of this population. Therefore, they concluded that both diets are effective primary therapies for active CD but do not influence long-term outcomes^73^. A third prospective randomized clinical trial also included 30 patients with active CD, with a mean CDAI of 301 (SES-CD 32), who would otherwise have been treated with steroids. The patients were randomized to receive an ED (n=16) or a PD (n=14) for 4 weeks. Clinical remission occurred in 10 of 15 patients on the ED, compared with 11 of 15 on the PD. Despite clinical improvement, nutritional therapy did not change nutritional status or various laboratory indices over 30 days. Therefore, polymeric formulas do not appear to offer an effective therapeutic alternative to elemental formulas in patients with acute flares of CD^74^.

### Partial enteral nutrition

**Recommendation**

Partial enteral nutrition (PEN) (≥900 kcal/day formula), when combined with pharmacotherapy, may help maintain remission and improve nutritional status in patients with luminal CD^75^ ^-^ ^78^.

**Consensus: 95.24%**

**Low quality of evidence**

Multiple studies have evaluated PEN as an adjunct to medical therapy for maintaining remission in luminal CD. The PEN accounted for 35-50% of estimated energy requirements, using a formula (polymeric, semi-elemental, or elemental). A cohort study including 102 patients on infliximab maintenance therapy showed that those receiving >900 kcal/day of PEN had a higher cumulative remission rate compared to patients without enteral supplementation (*P*=0.009)^75^. A prospective study of 56 post-induction CD patients (32 PEN + 24 controls) found lower endoscopic activity scores (1.25±0.25 vs 2.00±0.26; *P*=0.04), higher albumin levels (*P*=0.04), and greater BMI (*P*=0.03) at 12 months in the PEN group^77^. A randomized controlled trial with 51 patients in remission compared a half-elemental diet (900-1200 kcal/day) versus a free diet. The relapse rate was significantly lower with the half-elemental diet (34.6% vs 64.0%; HR 0.40, 95%CI 0.16-0.98) over a mean follow-up of 11.9 months^75^. Similarly, a longitudinal study of 39 CD patients in remission found that oral nutritional supplementation maintained remission in 48% of patients, compared with 22% on a regular diet (*P*<0.0003), with stable CDAI and CRP but improved body weight and BMI^78^. Overall, these findings suggest that PEN, when combined with pharmacotherapy (especially infliximab), supports remission maintenance, improves nutritional status, and may reduce the risk of relapse in luminal CD. In contrast to CD UC, dietary evidence in UC is more limited.

**FIGURE 1.**
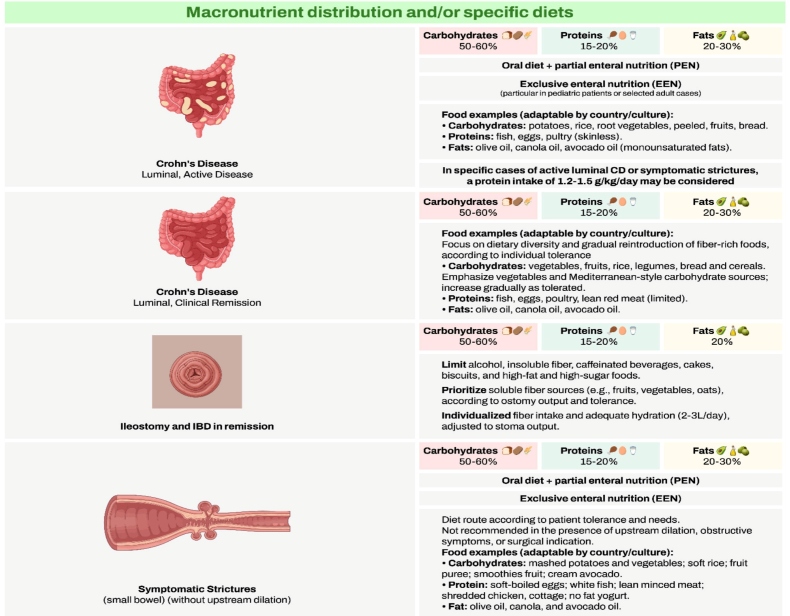
Practical nutritional guidance for the management of Crohn’s disease according to different clinical manifestations - Expert opinion. Overview of suggested macronutrient distribution and dietary patterns across different Crohn’s disease phenotypes during active disease and clinical remission, including special clinical scenarios such as ileostomy and symptomatic small-bowel strictures without upstream dilation. Recommendations are intended as general guidance and should be individualized based on disease phenotype, nutritional status, tolerance, and cultural context. Dietary recommendations should complement, not replace, pharmacological therapy. Note: These recommendations reflect expert opinion based on available evidence and clinical experience and are intended to support, rather than replace, individualized nutritional assessment. ©2026 GEDIIB. Ilustração desenvolvida exclusivamente para o Consenso de Nutrição do GEDIIB. Todos os direitos reservados.

**FIGURE 2.**
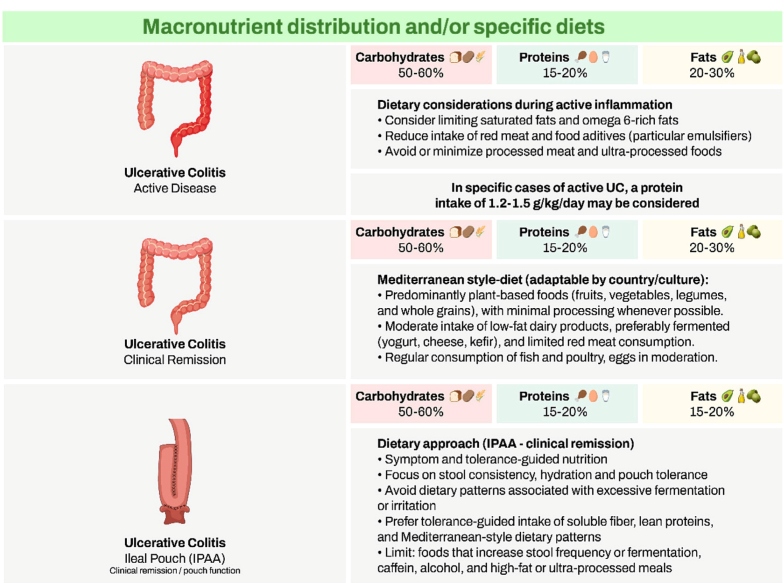
Practical nutritional guidance for the management of ulcerative colitis according to different clinical manifestations - Expert opinion.Overview of suggested macronutrient distribution and dietary patterns across different ulcerative colitis phenotypes during active disease and clinical remission, including patients with ileal pouch-anal anastomosis (IPAA). Recommendations are intended as general guidance and should be individualized based on disease phenotype, pouch function, nutritional status, tolerance, and cultural context. Dietary recommendations should complement, not replace, pharmacological therapy. Note: These recommendations reflect expert opinion based on available evidence and clinical experience and are intended to support, rather than replace, individualized nutritional assessment. © 2026 GEDIIB. Ilustração desenvolvida exclusivamente para o Consenso de Nutrição do GEDIIB. Todos os direitos reservados.

Taken together, FIGURES 1 AND 2 delineate the nutritional management strategies for the two distinct IBD phenotypes, stratified by active and quiescent disease phases, providing a standardized framework for personalized clinical decision-making^51^ ^,^ ^79^ ^-^ ^84^.

### Micronutrient supplementation

### Iron

**Recommendations**

**Intravenous iron is preferred as first-line therapy for patients with clinically active IBD, moderate-to-severe anemia, or intolerance to oral iron, given its greater efficacy in restoring hemoglobin levels and iron stores, as well as better tolerability** ^85^ ^-^ ^88^ **.**

The goal of iron supplementation is to normalize hemoglobin and iron stores, including ferritin and transferrin saturation^87^.

**Expert Opinion**

During active intestinal inflammation, hepcidin levels increase, inhibiting ferroportin (the main iron exporter in enterocytes) and reducing intestinal iron absorption and systemic iron availability. This mechanism results in functional iron deficiency despite adequate iron intake^89^ ^-^ ^92^.

**Consensus: 95.24%**

**Moderate quality of evidence**

Anemia, such as iron-deficiency, is common in IBD because mucosal inflammation causes blood loss and, less frequently, iron malabsorption, requiring oral or intravenous iron therapy. Current guidelines suggest that intravenous iron should be used as first-line therapy only for patients with active diseases, moderate-to-severe anemia, or intolerance to oral iron^85^ ^,^ ^93^. Five studies reported data on responding patients. Four studies defined “response” as an increase in hemoglobin of at least 2g/dL^86^ ^-^ ^88^ ^,^ ^94^. Ferric maltol achieved clinically relevant increases in hemoglobin but did not demonstrate non-inferiority to ferric carboxymaltose at week 12^85^ ^,^ ^94^ ^.^ Both treatments had comparable long-term effectiveness for hemoglobin and ferritin over 52 weeks and were well tolerated^95^ ^.^ Another study showed an increase in hemoglobin for both administration routes (median increase of 0.25 g/L in the intravenous group vs 0.21 g/L in the oral group); only iron sucrose increased serum ferritin concentrations^88^ ^.^ Regarding adverse events between intravenous and oral iron preparations, intravenous iron appears to be safer. For serious adverse events (SAEs), only Howaldt et al found a significant difference, with more SAEs reported in the oral iron group^95^. No statistically significant differences were noted in other studies^86^ ^,^ ^88^ ^,^ ^95^. Two studies reported higher discontinuation rates in the oral iron group^87^ ^,^ ^95^. Simultaneously, cobalamin and folate deficiencies increase the risk of macrocytosis and may be investigated in patients with a high Mean Corpuscular Volume (MCV). Serum levels of vitamin B12 and folate should be measured annually in patients with macrocytosis and in patients with small bowel disease or who have undergone resection. In these scenarios, closer monitoring is necessary^93^.

### Calcium and 25 (OH) vitamin D

**Recommendation**

Evidence does not confirm that serum calcium or 25(OH) vitamin D levels directly prevent bone loss in IBD. However, monitoring and correcting vitamin D deficiency are advised, particularly in steroid-treated patients with IBD^96^.

**Consensus: 85.71%**

**Very low quality of evidence**

Evidence for addressing these two questions remains limited. A multivariate analysis found there was no significant correlation between calcium and vitamin D levels and bone loss (OR=0.98; 95%CI 0.827-1.155; *P*=0.259). However, menopause (OR=8.532; 95%CI 3.356-55.633; *P*<0.001) and corticosteroid use (OR=2.73; 95%CI 1.143-6.236; *P*=0.012) were identified as significant risk factors for bone loss in IBD patients, whereas higher albumin levels were considered protective^96^. Given that most available studies are cross-sectional, establishing cause-and-effect relationships remains challenging. Furthermore, evidence focusing explicitly on clinical fractures as a primary outcome is lacking.

### Preoperative nutritional therapy in IBD

### Oral Nutritional supplements (ONS) or EEN

**Recommendations**

Preoperative enteral nutrition, including EEN or ONS, should be considered in surgical patients with IBD who are unable to meet nutritional requirements through oral intake, as it may reduce postoperative complications^97^ ^,^ ^98^.

However, evidence supporting the impact of preoperative enteral nutrition on broader outcomes (such as length of hospital stay, need for reoperation, or overall postoperative morbidity) remains limited and inconsistent^98^ ^-^ ^102^.

**Consensus: 85.71%**

**Low quality of evidence**

In the retrospective cohort study, nutritionally optimized patients experienced fewer overall complications, surgical, non-surgical, and infectious events^97^. In another prospective study, patients who received preoperative enteral nutrition compared with those undergoing upfront surgery had similar postoperative outcomes (overall morbidity rates, rates of intra-abdominal sepsis and severe complications, reoperation rates, postoperative drainage, and length of hospital stay)^99^. A study also evaluated the preoperative optimization strategy: 45 CD patients received EEN for at least 4 weeks, whereas 75 CD patients did not. Patients who received EEN experienced fewer postoperative complications, surgical site infections, and a lower comprehensive complication index (P<0.05). However, after categorizing mild or major complications and the relation to postoperative length of stay, no difference between the two groups was observed^100^. For clinical outcomes, including clinical and endoscopic remission, most studies found no significant difference between groups, especially after 6 months^99^ ^-^ ^104^. Preoperative enteral nutrition was associated with lower infectious and non-infectious complications in most studies^98^ ^,^ ^99^ ^,^ ^101^ ^,^ ^102^. However, results were inconsistent across studies regarding immediate postoperative outcomes, such as time to resumption of oral intake and length of hospital stay^99^ ^,^ ^100^ ^,^ ^103^.

### Total parenteral nutrition (TPN)

**Recommendation**

Preoperative TPN does not appear to reduce postoperative complications and should be reserved for patients who are unable to tolerate or receive enteral nutrition^104^ ^,^ ^105^.

**Consensus: 90.48%**

**Low quality of evidence**

The cohort study showed no significant differences in overall complication rates between the TPN and non-TPN groups (29.1% vs 26.9%; *P*=0.78). Infectious complications were observed in 18.2% of patients in the TPN group compared to 12.3% in the non-TPN group (*P*=0.34), while non-infectious complications occurred in 14.5% vs 16.9%, respectively (*P*=0.71)^105^. Another longitudinal study divided patients into two groups: those who received preoperative TPN (n=40) and those who did not (n=129). The duration of TPN administration was 12.9 days (±9.2). The incidence of infectious complications, including wound infections and intra-abdominal collections, was comparable between groups (7.5% vs 9.3%; *P*=0.600). Non-infectious complications-such as anastomotic leaks, wound dehiscence, intestinal obstruction, enterocutaneous fistula, and incisional hernia showed no significant difference (17.5% vs 15.5%; *P*=0.630)^104^. There were no significant differences in infectious or non-infectious complications between patients who received preoperative parenteral nutrition and those who did not in both studies analyzed. No studies specifically analyze patients who received parenteral nutrition due to enteral nutrition intolerance. The current evidence is predominantly focused on CD, with limited data available for UC.

### Early/enhanced recovery after surgery (ERAS protocol)

**Recommendations**

Enhanced Recovery After Surgery (ERAS) protocols are associated with shorter postoperative hospital stays and earlier recovery of gastrointestinal function and appear to be safe in elective IBD surgery. Available data include different surgical procedures, but the evidence is more robust for laparoscopic ileocecal resection^106^ ^,^ ^107^.

Further studies are required to clarify the impact of ERAS protocols on major postoperative and long-term outcomes.

**Consensus: 90.48%**

**Low quality of evidence**

In a retrospective study, patients in the ERAS protocol had a shorter median length of stay (6 [5-8.5] vs 8 [7-10] days; *P*<0.001), earlier initiation of unrestricted oral intake (2 [1-3.5] vs 4.5 [2-6.5] days; *P*<0.001), and earlier first bowel movement (1 [1-2] vs 2 [2-3] days; *P*<0.001). There were no significant differences between the groups in the rates of major complications (Clavien-Dindo ≥ IIIb), postoperative fistula, postoperative bleeding, or readmission within 90 days^106^. In a randomized, consecutive cohort study of 32 patients with CD undergoing laparoscopic ileocecal resection, patients in the ERAS protocol showed a significantly earlier return of bowel function, with shorter times to first flatulence and first bowel movement. In addition to initiating liquid and semi-fluid diets, hospital costs were lower, and postoperative hospitalization was shorter (*P*<0.001). For other outcomes - including complications (grade I and grade II-IV), reoperations, readmission within 30 days, postoperative pain, hospital mortality, and infectious complications within 30 days - no significant differences were observed between the groups. In summary, the ERAS protocol in CD patients facilitates accelerated gastrointestinal recovery and reduces hospital stay, without increasing the risk of major postoperative complications or readmissions. An ERAS perioperative care program is a safe and effective treatment combination for patients with CD who require ileocecal resection^107^.

### Postoperative outcomes in IBD

### Malnutrition (undernutrition and overnutrition)

**Recommendation**

Current evidence does not support the use of BMI or body weight alone as predictors of poor postoperative outcomes in IBD surgery^108^ ^,^ ^109^.

**Expert opinion**

Obesity, low BMI, and hypoalbuminemia are associated with increased postoperative morbidity and mortality in patients with CD and UC, particularly when considered in combination with disease activity and other clinical risk factors^110^ ^,^ ^111^.

**Consensus: 95.48%**

**Low quality of evidence**

A retrospective cohort study evaluated the influence of body weight on surgical outcomes in patients with IBD, including underweight (n=34), normal weight (n=187), overweight (n=105), and obese (n=65). Intraoperative complication rates were comparable across all groups, with no significant differences observed in overall complications (*P*=0.561), bleeding (*P*=0.400), fecal spillage (*P*=0.518), visceral injury (*P*=0.400), or hospital length of stay (*P*=0.171). Likewise, postoperative outcomes, including cardiac, pulmonary, renal, and infectious complications, anastomotic leaks, small bowel obstruction, reoperation, and wound issues, showed no significant differences^108^. Another retrospective cohort analysis using propensity score matching was performed to compare surgical outcomes between obese (n=659) and non-obese (n=659) patients. While in-hospital mortality rates remained low and statistically similar (*P*=0.54), obese patients had a significantly higher rate of postoperative intensive care unit (ICU) admissions (*P*=0.01) and overall complications (*P*=0.01). Notably, there were no significant differences in respiratory, cardiovascular, infectious, or wound-related complications or post-procedural shock. However, gastrointestinal and genitourinary complications were more frequent among obese patients. Despite these findings, the average hospital stay was nearly identical between groups (*P*=0.77)^109^.

### Sarcopenia

**Recommendation**

Sarcopenia appears to be an independent risk factor for postoperative complications in patients with CD and should be considered in the preoperative risk assessment^112^ ^-^ ^114^.

**Consensus 90.48%**

**Low quality of evidence**

A prospective study of 137 patients with CD that analyzed surgical outcomes and which nutritional indicators serve as risk factors for postoperative complications, concluded that sarcopenia was an independent risk factor for postoperative complications (OR 2.85; 95%CI 1.13-7.16; *P*=0.03)^112^. Patients with sarcopenia had longer total and postoperative hospital stays and more complications than non-sarcopenic patients (*P*=0.049). Other parameters, including readmission within 30 days, reoperation, ICU admission, and death, did not differ between the groups. Multivariate analysis demonstrated that sarcopenia was a risk factor for postoperative complications (OR=3.974, 95%CI 1.171-13.489; *P*=0.027)^113^. Another important aspect was that Clavien-Dindo grade 3 to 5 complications occurred more frequently in patients with sarcopenia (*P*=0.027). Multivariate analysis defined sarcopenia as a risk factor for complications (CD ≥3) (OR=9.24, 95%CI 1.10-77.50; *P*=0.04)^114^. In summary, sarcopenia is consistently associated with higher rates of postoperative complications across the analyzed studies. See also recommendation 3.2.

### Short Bowel Syndrome and GLP-2 analog

**Recommendation**

GLP-2 analogues (evidence limited to teduglutide) may be considered in selected CD patients with Short Bowel Syndrome or Intestinal Failure who remain dependent on parenteral nutrition despite optimal management; however, evidence regarding efficacy and safety remains limited^115^.

**Consensus: 90.48%**

**Low quality of evidence**

Few longitudinal studies have evaluated GLP-2 analogs in Short Bowel Syndrome (SBS). A cohort study followed 32 patients with CD and SBS receiving teduglutide (a GLP-2 analog). Outcomes were compared before and after initiation of the medication. All patients require Parenteral Support (PS) or intravenous fluids (IVF). At baseline, 16 patients received both PN and IVF, 7 received PN alone, and 9 received IVF alone. Following initiation of teduglutide, the number of patients requiring PN decreased from 23 to 14. Twenty-six of 32 patients achieved the primary outcome of ≥20% reduction in PS, with eight patients successfully wearing off all support (*P*<0.01). While teduglutide may help reduce dependence on PS, its tolerability remains a consideration^115^.

## DISCUSSION

Over the past three years, nutritional guidelines for IBD have significantly evolved, shifting from merely supportive to becoming a key part of treatment, especially in CD. However, there remains limited evidence on nutritional treatment in UC, and additional clinical trials and long-term studies are necessary.

Currently, nutritional management is guided by three major international organizations and, in Brazil, by GEDIIB. Among the most recent guidelines and consensus, the following are notable:

The European Society for Clinical Nutrition and Metabolism (ESPEN)^51^ is the leading authority on clinical nutrition. Its recommendations focus on overall nutritional status and on mandatory screening for malnutrition at diagnosis and periodically, even with normal or high BMI, due to the risk of sarcopenia. Protein requirements should be increased during the active phase (1.2-1.5 g/kg/day in adults). Micronutrients should be monitored annually for iron, vitamin D, B12, and folic acid.

The International Organization for the Study of IBD (IOIBD) recommendations focused on specific dietary components^116^. Fats should be reduced, with saturated fats and trans fats avoided in both diseases. In UC, it is recommended to increase the intake of Omega-3 (through diet). In UC, it is prudent to reduce consumption of red and processed meat to avoid relapses. In CD, restricting ultra-processed foods is recommended, and red meat is not mandatory. Additionally, a drastic reduction of emulsifiers (such as carboxymethylcellulose and polysorbate 80) and artificial sweeteners, which can alter the intestinal barrier, must be adopted.

The European Crohn’s and Colitis Organization (ECCO) recently issued updates on “Dietary Management”^40^: Dietary Pattern: A plant-based diet, high in fruits and vegetables (adjusting texture if strictures are present), and low in ultra-processed foods is recommended. Remission Induction: EEN remains the primary treatment for pediatric CD. For adults, CDED combined with PEN has gained popularity as an effective alternative. There is no evidence to support restrictive diets (such as gluten-free or lactose-free) for everyone unless there is a proven intolerance.

The nutrition management of IBD - a consensus from the Brazilian Organization for Crohn’s and Colitis (GEDIIB) - does not aim to cover all nutritional aspects of IBD treatment but focuses on the main ones. It emphasizes the importance of specialized nutritionists at all stages of IBD patient care, adapting international recommendations to our context, and prioritizing whole foods and regional agriculture.

### Future directions

It is essential to include specialist dietetic input in both clinical care and IBD research. In accordance with international nutrition guidelines^51^ ^,^ ^117^ and consensus in IBD^40^, it is strongly recommended that a nutritionist with specialized, experienced expertise in IBD management perform nutritional assessment. This is particularly critical regarding restrictive diets, which pose significant nutritional risk and may trigger disordered eating patterns and psychosocial distress.

Furthermore, IBD patients require individualized nutritional therapy tailored to their clinical phenotype, disease activity, and remission status. In Brazil, meeting this need depends on the professional development of specialized IBD nutritionists and the strategic expansion of nutrition-focused research to provide local evidence-based care.

## Data Availability

not applicable - the study did not use research data
